# Integrating Surgery and Ablative Therapies for the Management of Multiple Primary Lung Cancer: A Systematic Review

**DOI:** 10.3390/cancers17223699

**Published:** 2025-11-19

**Authors:** Zhenghao Dong, Cheng Shen, Jingwen Zhang, Jian Zhou, Xiang Lin, Beinuo Wang, Hu Liao

**Affiliations:** 1 Department of Thoracic Surgery, West China Hospital, Sichuan University, Chengdu 610041, China; 2 West China School of Medicine, Sichuan University, Chengdu 610041, China

**Keywords:** multiple primary lung cancer, ablation, ablative therapies, surgery, integration therapy, case report

## Abstract

***Background:*** Multiple primary lung cancer (MPLC) presents clinical challenges due to its biological complexity. While lobectomy remains standard, limited resection and localized ablation offer comparable efficacy. This systematic review evaluates the safety and efficacy of combining surgical and ablative therapies for MPLC. ***Methods:*** A comprehensive search of PubMed, Embase, and Web of Science (January 2000–2025) identified studies involving MPLC patients treated with both surgery and ablation, either concurrently or sequentially. Data on ablation efficacy, adverse events, and prognosis were extracted. A meta-analysis was performed when data pooling was appropriate. The methodological quality and risk of bias of the included studies were assessed using the MINORS and ROBINS-I tools. Publication bias was evaluated through funnel plots and Egger’s linear regression test. Furthermore, one case report on combination therapy was also included. ***Results:*** A total of nine studies met the inclusion criteria and were included in the final analysis. All reported a 100% technical success rate for ablation, efficacy rates exceeding 70%, and adverse event rates ranging from 5.0% to 26.7%. Due to significant heterogeneity among studies, a random-effects model was applied. The meta-analysis yielded a pooled ablation efficacy rate of 97.11% (95% CI: 85.81–100.00%) and a pooled adverse event rate of 14.23% (95% CI: 8.07–20.38%), indicating favorable safety and efficacy of the combined therapy. ***Conclusions:*** The integration of surgical and ablative therapies offers a safe and effective strategy for managing MPLC and supports a potential paradigm shift from single-modality treatment toward a more personalized, organ-preserving, and patient-centered approach.

## Simple Summary

Multiple primary lung cancer (MPLC) is becoming more common with increased use of chest imaging. Treating patients with many lung tumors is challenging, as traditional surgery may not be suitable or necessary for all lesions. Ablation, a minimally invasive technique that destroys tumors using heat or cold, has shown promise for managing selected lesions. This review explores the use of combining surgery and ablation to treat MPLC. Based on the available evidence, this integrated approach appears to be safe and effective, with promising success rates and relatively few complications. It allows for preservation of more lung tissue and tailoring of treatment to individual patient needs. Our findings suggest that this strategy may offer a valuable treatment option, potentially preserving more lung function compared to extensive surgery alone, especially for patients with multiple or hard-to-reach tumors. These insights could inform future treatment guidelines and support more personalized care for patients with complex lung cancer.

## 1. Introduction

Lung cancer remains one of the most prevalent and lethal malignancies both in China and globally [1,2,3]. With the widespread adoption of chest computed tomography (CT) and increasing life expectancy, the incidence of MPLC has been rising [4,5,6,7]. Clinical studies estimate that the incidence of MPLC ranges from 1% to 20%, with a higher prevalence observed among women and non-smokers [8,9,10,11,12]. Notably, since the onset of the COVID-19 pandemic in 2019, there has been a significant increase in the number of individuals undergoing lung CT screening, leading to a sharp rise in the detection of multiple lung nodules [13,14,15,16,17]. The first case of MPLC was reported by Beyreuther in 1924, marking the initial recognition of this condition in clinical practice [18,19]. MPLC is characterized by the presence of two or more independent primary cancerous lesions in the lungs, which arise either simultaneously or sequentially from distinct anatomical origins. Clinically, MPLC is further classified based on the interval between diagnoses into synchronous MPLC (sMPLC) and metachronous MPLC (mMPLC) [20,21,22]. Unlike conventional lung cancer, MPLC exhibits distinct clinical and genetic characteristics [23,24,25]. Its multiplicity and inter-focal heterogeneity present considerable challenges in diagnosis and treatment [26]. Despite ongoing research, there is currently no standardized treatment protocol for MPLC.

At the time of initial diagnosis, most patients with MPLC present with multiple intrapulmonary ground-glass opacities (GGOs) on imaging, with surgical resection being the primary treatment modality in the early stages of the disease [27,28]. However, the multiplicity and diffuse distribution of MPLC lesions pose significant challenges for complete surgical resection in clinical practice. Complete removal of all nodules often requires multiple complex segmentectomies or even bilateral surgeries, which substantially increase intraoperative risks and the likelihood of postoperative complications. Furthermore, such extensive surgical interventions may significantly impair postoperative quality of life by reducing lung function and reserve capacity. Notably, in most cases, complete resection of all lesions is not considered necessary [29]. As a result, non-surgical ablation therapy has emerged as a viable alternative for patients with early-stage MPLC who are either ineligible for surgery or decline surgical treatment [30].

Ablation therapy employs heating or freezing techniques to induce irreversible damage or coagulative necrosis in localized tumor tissues, thereby effectively eradicating cancer cells [31,32,33]. The three primary modalities of lung ablation therapy are radiofrequency ablation (RFA), microwave ablation (MWA), and cryoablation (CA) [34,35]. RFA and MWA utilize thermal energy to destroy tumor cells, whereas CA achieves its effects through repeated cycles of freezing and thawing [36,37]. For patients with MPLC who have severe cardiopulmonary comorbidities or decline surgery, ablation techniques are strongly recommended. This approach offers several advantages, including high repeatability, the ability to treat multiple lesions either simultaneously or in a staged manner, on the same or opposite lung, minimal postoperative complications, and cost-effectiveness [38]. With continuous advancements in surgical techniques and medical equipment, ablation therapy can be performed as a standalone treatment or in combination with surgery [39]. In recent years, the integration of surgical and ablation therapies has been increasingly utilized in the management of MPLC, demonstrating promising clinical outcomes.

Given these circumstances, the primary objective of this review was to evaluate the existing results of safety, feasibility and efficacy of this combined therapy in the management of MPLC. This review also included a case report of a patient with multiple pulmonary nodules who underwent combined surgical resection and ablation.

## 2. Methods

This systematic review and meta-analysis was conducted in accordance with the Preferred Reporting Items for Systematic Reviews and Meta-Analyses (PRISMA) 2020 and has been reported in line with Assessing the methodological quality of systematic reviews (AMSTAR 2) Guidelines [40,41].

This review was registered with PROSPERO (Unique Identifying Number: CRD420251038693).

### 2.1. Search Strategy

**Figure 1:**
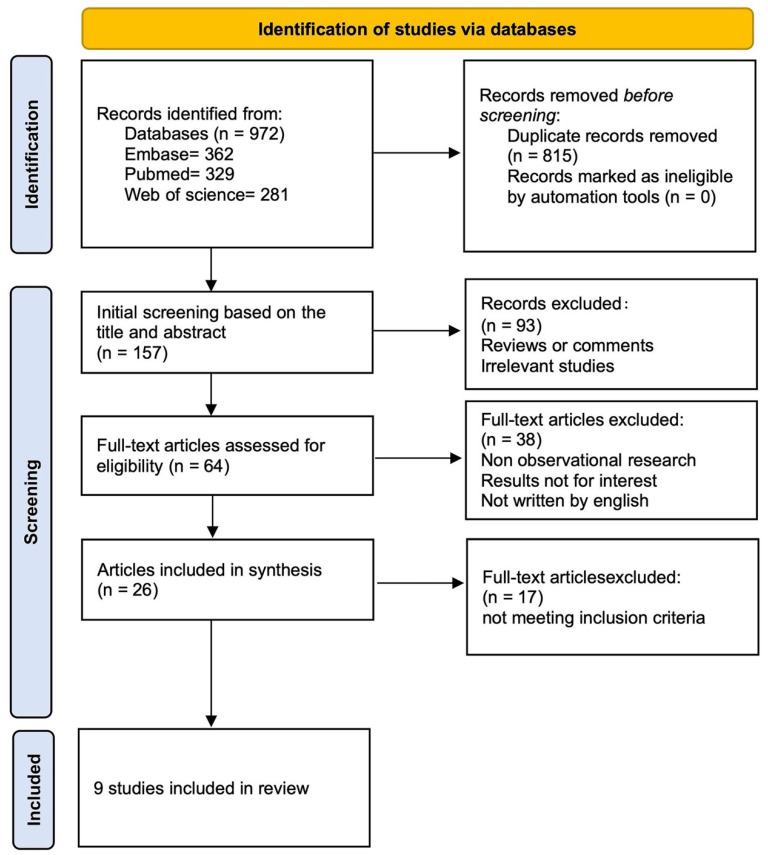
Flow chart of literature search strategies.

A systematic search was conducted across three electronic databases—PubMed, Embase, and Web of Science—covering the period from January 2000 to January 2025. To ensure optimal retrieval, both database-specific controlled vocabulary (e.g., MeSH terms) and free-text keywords were employed. The MeSH terms included “Multiple Pulmonary Nodules”, “Lung Neoplasms”, “Ablation techniques” and “Radiofrequency Ablation”. Additionally, a manual search of key reference lists was performed to identify further eligible studies (see Supplementary Materials for the full search strategy). Two independent researchers (Z.D. and J.Z.) screened titles, abstracts, and full texts and extracted data based on standardized criteria. All search results were merged, and duplicates were removed prior to selection. A unified search strategy was applied, and studies were categorized accordingly for bias assessment and data extraction. The search and selection process is detailed in Figure 1.

Articles included in this review met the following eligibility criteria: (1) The study was an original research article; (2) Patients were diagnosed with multifocal lung lesions; (3) Patients underwent surgical treatment combined with ablation therapy, either simultaneously or sequentially; (4) The study reported at least one specific outcome related to the safety and/or efficacy of the combined treatment.

The following exclusion criteria were applied: (1) Patients with a history of malignancies in other organ systems or those with lung metastases; (2) Case reports and studies lacking original data, such as reviews, comments, or editorials; (3) Other conditions deemed unsuitable for inclusion.

The initial screening was conducted based on titles and abstracts, with independent evaluation of the retrieved literature. Abstracts that lacked sufficient information were further assessed through full-text review. Subsequently, two researchers (Z.D. and C.S.) independently reviewed the full-text articles and determined their eligibility for inclusion.

### 2.2. Data Extraction and Quality Assessment

Data extraction was conducted by two researchers (Z.D. and J.Z.) following standardized information extraction protocols. The type of study, year, patients, treatment, safety and efficacy of ablation, incidence of adverse events, and patient prognosis were extracted for the included article. The results were subsequently reviewed by a senior researcher (C.S.) for accuracy and consistency. Two researchers (Z.D. and C.S.) independently assessed the methodological quality and risk of bias of the included studies using the MINORS and ROBINS-I tools [42,43].

### 2.3. Statistical Analysis and Publication Bias

Data from the included studies were analyzed using a random-effects model with the R “meta” package to evaluate the safety and efficiency of the combined therapy. Pooled estimates were calculated as event rates with their 95% confidence intervals (CIs). Heterogeneity across studies was assessed using Cochran’s Q test and the *I*^2^ statistic. *I*^2^ values of 25%, 50%, and 75% were interpreted as indicating low, moderate, and high heterogeneity, respectively, representing the proportion of total variation attributable to heterogeneity rather than chance [44]. A Q test *p*-value of <0.05 was considered indicative of significant heterogeneity [45]. Potential publication bias was evaluated using funnel plots and Egger’s test [46]. Given the limited number of studies included, subgroup and sensitivity analyses were not performed.

All statistical analyses were conducted using R software (version 4.3.2). Statistical significance was defined as a two-sided *p*-value < 0.05.

## 3. Results

### 3.1. Study Selection

A total of 972 studies were identified through database searches up to January 2025. After removing duplicates, 157 records remained for screening. Of these, 93 were excluded based on study type. Full-text assessment of the remaining 64 articles led to the exclusion of 55 that did not meet the eligibility criteria. Ultimately, 9 studies were included in the review, comprising 3 prospective and 6 retrospective studies.

### 3.2. The Characteristics of Included Studies

Bao et al. [47] conducted a prospective study evaluating the safety, feasibility, and technical efficacy of electromagnetic navigation bronchoscopy (ENB)-guided microwave ablation (MWA). Zeng et al. [48] assessed the short-term safety and effectiveness of ENB-guided MWA in patients with multiple pulmonary nodules (MPNs). Liu et al. [49] reviewed surgical and ablative approaches for multifocal GGO-type adenocarcinoma. Harrison et al. [50] described their technique and presented a retrospective case series of 4 patients who underwent image-guided combined ablation and resection in thoracic surgery (iCART). Xie et al. [51] reported 19 MWA sessions performed on 14 tumors under navigation bronchoscopy. Zhou et al. [52] presented real-world data on a “Surgery+X” strategy for early-stage sMPLC. Qu et al. [53] investigated the safety and feasibility of combining ENB-guided MWA with uniportal video-assisted thoracoscopic surgery (VATS) for multiple GGOs. Zarogoulidis et al. [54] utilized radial endobronchial ultrasound (rEBUS), C-arm fluoroscopy, and Archimedes navigation for biopsy and applied radiofrequency ablation for central pulmonary nodules. Shan et al. [55] examined the safety and feasibility of CT-guided thermal ablation combined with intraoperative biopsy for multiple pulmonary nodules. The detailed characteristics of the included studies are summarized in Table 1.

### 3.3. Quality and Risk of Bias Within Studies

MINORS scores ranged from 10 to 14, indicating moderate methodological quality (Supplementary Table S1). According to the ROBINS-I tool, the overall risk of bias was also judged to be moderate (Supplementary Figure S1). Most studies were judged to have a high risk of bias due to insufficient reporting or adjustment of baseline characteristics, a common limitation in single-arm surgical trials where confounding factors are difficult to control. Zeng et al. [48] and Zarogoulidis et al. [54] did not report baseline data, while Bao et al. [47] and Xie et al. [51] may have lacked proper adjustment. Other studies documented baseline characteristics at enrollment.

### 3.4. The Ablation Safety and Efficacy of Included Studies

Ablation is generally considered a safe procedure when performed by experienced medical professionals. Across all included studies in this review, both the technical success rate of combination therapy and the safety rate of ablation were consistently reported as 100.00%.

The efficacy of ablation is primarily assessed by its ability to eradicate malignant tissue. Clinically effective ablation is defined as the “complete ablation” of a macroscopic tumor, as evidenced by post-procedural imaging showing the treated tumor encompassed by a larger solid or GGO area. Successful ablation can result in substantial tumor size reduction or complete tumor eradication, thereby contributing to disease control. Seven studies reported the efficacy of ablation, with four demonstrating a 100.00% ablation success rate. In these studies, all treated lesions exhibited a complete response, with no evidence of progression during follow-up. Bao et al. [47] reported that CT scans performed during the first postoperative week confirmed technical effectiveness in 11 out of 15 nodules (73.33%). Similarly, in Xie et al.’s study [51], among 14 treated tumors, 11 were completely ablated, one exhibited incomplete ablation, and two showed local progression post-treatment, resulting in a complete ablation rate of 78.57% (11/14). For detailed information regarding ablation safety and efficacy, refer to Table 2.

### 3.5. Ablation-Related Adverse Events and Complications

Although ablation is generally regarded as a safe intervention, it is not without risk, particularly in patients with compromised pulmonary function or multiple lesions. The common adverse events and complications associated with pulmonary nodule ablation are summarized in Table 3.

Reported adverse events and complication rates in the included studies ranged from 5% to 26.7%. Detailed data are summarized in Table 2. Bao et al.’s study [47] observed hemoptysis in one patient with single lesion and complications in three of ten patients with multiple lesions, including air leakage, hemoptysis, and pulmonary infection. Similarly, Zeng et al. [48] reported complications such as pain (n = 5), pneumothorax (n = 2), subcutaneous emphysema (n = 2), and persistent cough (n = 6). Liu et al. [49] reported prolonged air leakage (>1 week) in two cases, chylothorax in one, and significant pleural effusion in one case. Zhou et al.’s study [52] documented only two instances of mild, asymptomatic pneumothorax among 43 ablated lesions. In Qu et al.’s study [53], only one patient developed pneumothorax and subcutaneous emphysema postoperatively, both of which resolved with symptomatic management. No other severe complications or surgery-related mortality were reported.

### 3.6. Meta-Analysis

**Figure 2:**
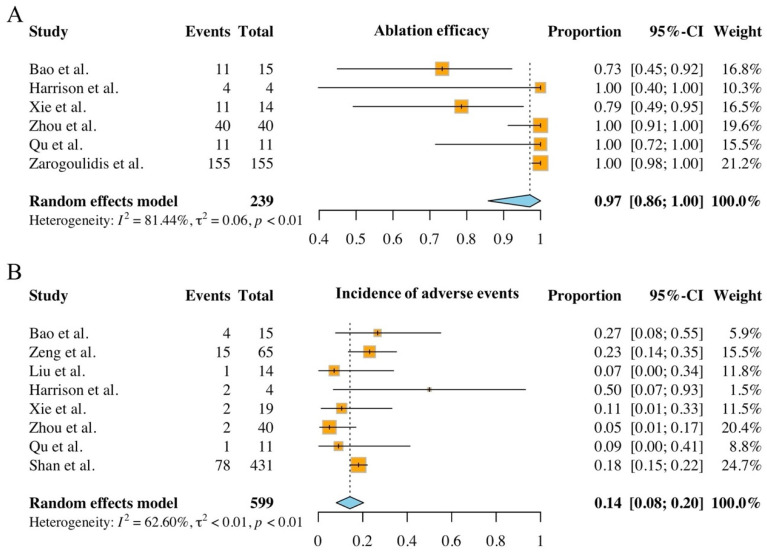
Meta-analysis of the efficacy (**A**) [47,50,51,52,53,54] and safety (**B**) [47,48,49,50,51,52,53,55] of combining surgical and ablative therapies for multiple primary lung cancers. The combined overall effect was estimated using random-effects models.

Of the nine studies included, six reported ablation efficacy and were incorporated into the meta-analysis. The pooled efficacy rate was 97.11% (95% CI: 85.81–100.00%), with substantial heterogeneity observed (Q = 26.93, *df* = 5, *p* < 0.001; *I*^2^ = 81.44%, 95% CI: 60.30–91.30%), justifying the use of a random-effects model (Figure 2A). Eight studies reported intraoperative or postoperative adverse events, also showing significant heterogeneity (Q = 18.72, *df* = 7, *p* = 0.001; *I*^2^ = 62.60%, 95% CI: 19.50–82.60%). The pooled incidence of adverse events was 14.23% (95% CI: 8.07–20.38%) (Figure 2B). Although the number of included studies limited the interpretability of funnel plots (Supplementary Figure S2), Egger’s tests detected no significant publication bias for either outcome (efficacy: *p* = 0.253; adverse events: *p* = 0.934).

## 4. Case Report

We report a 51-year-old female patient who underwent combined surgical and ablation treatment for multiple pulmonary nodules. Informed consent was obtained from the patient involved in this case.

### 4.1. Clinical History

She was admitted with incidentally detected multiple pulmonary nodules on routine physical examination on 17 October 2024. She had taken oral moxifloxacin for 7 days prior to admission without radiologic improvement, suggesting ineffective anti-inflammatory therapy. She was asymptomatic, with no history of smoking or environmental exposure, or family history of cancer. Her medical history included allergic asthma for over 30 years, managed with salmeterol inhalation. Surgical history included turbinate reduction and cesarean section over 20 years ago, and excision of a left breast nodule 3 years prior (benign pathology). She had no history of lung surgery or other significant comorbidities.

### 4.2. Preoperative Examinations

**Figure 3:**
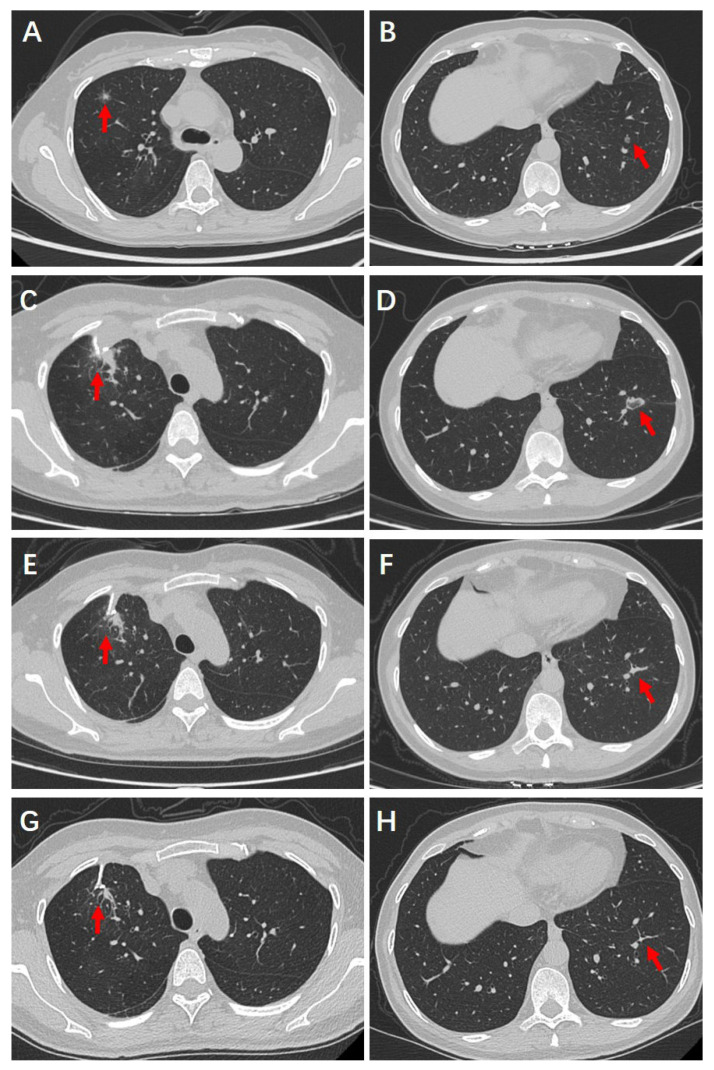
Pre-and postoperative High-Resolution Computed Tomography images of contralateral lesions managed by surgical resection and radiofrequency ablation. The primary part (right upper lobe)-solid nodule with spiculated margins underwent resection, while the contralateral lesion (left lower lobe) was ablated. Follow-up demonstrated progressive shrinkage and fibrosis of the lesions. (**A**) Resected lesion, preoperative. (**B**) Ablated lesion, preoperative. (**C**) One month after resection. (**D**) One month after ablation. (**E**) Three months after resection. (**F**) Three months after ablation. (**G**) Six months after resection. (**H**) Six months after ablation. The arrows indicate the location of the lesions and the changes observed during postoperative follow-up.

On admission, physical examination revealed normal breath sounds bilaterally. Preoperative cardiopulmonary and thyroid function tests were within normal limits, as were routine laboratory evaluations, including hematologic, coagulation, hepatic, and renal panels. Chest CT identified a 1.2 × 1.2 cm part-solid nodule with ill-defined margins, spiculations, and air bronchograms in the anterior segment of the right upper lobe (Figure 3A). Another 0.6 cm nodule was noted in the outer basal segment of the left lower lobe (Figure 3B). Cranial and abdominal CT scans were unremarkable, with no evidence of distant metastasis.

### 4.3. Surgical and Ablative Management

As imaging could not definitively characterize the pulmonary lesions, the patient was scheduled to undergo wedge resection of the right upper lobe lesion under single anesthesia in a hybrid operating room, combined with CT-guided radiofrequency ablation of the left lower lobe lesion. Intraoperatively, the right upper lobe nodule (1.2 × 1.2 cm) was located in the anterior segment, with no signs of visceral pleural retraction or parietal pleural invasion and was >2 cm from the carina. After resection, the patient was placed in the right lateral decubitus position for RFA. Under C-arm CT guidance, the left lower lobe nodule was localized, and the puncture path was planned. Forty watts power setting was applied, and ablation lasted 3 min, ensuring the needle passed through the lesion with a 0.5 cm margin. Post-ablation CT revealed slight collapse of the nodule with surrounding GGO measuring approximately 2 × 2.5 cm.

### 4.4. Postoperative Recovery and Follow-Up Outcomes

The patient was able to ambulate on postoperative day (POD) 1 without any notable discomfort. The chest drainage tube was removed on POD 2, with a total output of 165 milliliters, and she was discharged on POD 3. Postoperative histopathological biopsy confirmed that the resected lesion in the right lung was a minimally invasive adenocarcinoma. Follow-up chest CT scans at 1, 3, and 6 months postoperatively demonstrated progressive resolution of the residual tissue in the right upper lobe resection area (Figure 3C,E,G) and the ablation zone in the left lower lobe (Figure 3D,F,H). Notably, the ablated nodule in the left lower lobe had been completely resorbed and was no longer visible. The patient remains alive and under ongoing clinical follow-up.

## 5. Discussion

The systematic review of the included studies demonstrated that combined surgical and ablative approaches exhibits a favorable safety-efficacy profile for the treatment of MPLC. All included studies reported a 100.00% technical success rate for ablation, with a pooled efficacy rate of 97.11% (95% CI: 85.81–100.00%) and a low adverse events rate (pooled rate: 14.23%, 95% CI: 8.07–20.38%). These findings highlight the necessity of personalized therapeutic strategies to optimize clinical outcomes, emphasize their integration as a unified therapeutic model, which is particularly advantageous for patients with multiple or bilaterally distributed lesions who are unsuitable for extensive surgery.

Since Beyreuther’s initial report on multiple pulmonary lesions in 1924, MPLC has garnered increasing attention. Studies suggest that 6–8% of patients with non-small cell lung cancer (NSCLC) develop multifocal lesions during their disease course [56], while 1.5–3.0% of primary lung cancer patients eventually develop a second primary lung cancer, indicating a time-dependent cumulative effect on MPLC incidence [57,58]. The diagnostic criteria for MPLC were first established by Martini and Melamed in 1975, based on histological type and lesions distribution [59]. In 1995, Antakli proposed that for lesions of the same histological type, differences in DNA ploidy counts should be considered [60]. However, due to technical limitations at the time, its clinical application was limited. In 2003, the American College of Chest Physicians (ACCP) refined these criteria by emphasizing molecular genetic differences between lesions, contributing to the development of more comprehensive diagnostic guidelines for MPLC in clinical practice [61]. A critical challenge in MPLC diagnosis is its differentiation from intrapulmonary metastases (IPM), as these conditions require distinct treatment strategies and have significantly different prognoses [62,63,64]. Comprehensive histopathological assessments, including analyses of histological subtypes, cytological features, and genomic mutations, play a crucial role in distinguishing MPLC from IPM [65,66,67]. In most of the studies included in this review, the enrolled patients presented with multiple GGO, which are generally regarded as synchronous multiple primary adenocarcinomas rather than intrapulmonary metastases [68]. Nevertheless, explicit molecular or histopathological confirmation of clonal independence was not consistently reported across studies.

High-quality clinical research on MPLC treatment remains limited, with therapeutic strategies generally included surgical and non-surgical approaches. MPLC often manifests as multiple GGO, which are traditionally treated with surgical resection [69]. However, no standardized surgical approach has been established, and treatment strategies are generally determined based on lesion size, number, and location, and the patient’s overall preoperative condition. Although surgical intervention aims to achieve complete lesion removal, complete resection is not always necessary [29]. The extensive distribution and multiplicity of MPLC lesions often render single-stage resection impractical, as it may result in substantial loss of lung function and an increased risk of complications. While staged surgery mitigates immediate surgical risks, it is associated with higher medical costs, increased procedural complexity, and greater psychological and financial burdens on patients.

In contrast to two-stage surgical strategies, the combined surgery + ablation model offers a lung-parenchyma-sparing alternative. Two-stage surgery—typically involving sequential operations to resect multiple lesions—has the advantage of established surgical technique, systematic lymph node dissection, and clear pathological margins. However, it may entail multiple hospitalizations and anesthesia sessions, increased cumulative risk of complications, longer total recovery time, more extensive loss of lung function across operations, and greater patient burden (both psychological and economic). By comparison, the integrated surgery-ablation approach reduces the number of anesthesia events, shortens overall hospital stay, preserves more healthy lung tissue, and lessens procedural trauma, making it particularly suitable for patients with limited pulmonary reserve or bilateral lesions. That said, potential limitations of the combined strategy remain: the ablation component may have less robust long-term outcome data than surgical resection, margin status may be less definitively assessed than in open surgery, and specialized interventional expertise is required.

Non-surgical treatments for MPLC include stereotactic body radiotherapy (SBRT), targeted therapy, immunotherapy, and ablation therapy. SBRT, as a widely adopted alternative for early-stage NSCLC in medically inoperable patients, merits specific consideration in the context of MPLC. Studies in MPLC settings have shown high two-year local control rates with minimal high-grade toxicity [70,71]. Evidence therefore supports SBRT as an appropriate option when surgery or ablation are not feasible. However, SBRT also has notable limitations: it does not allow for systematic pathological lymph-node assessment, treating multiple closely spaced lesions may be constrained by cumulative lung dose limits, and there remains a risk of radiation-related pneumonitis or fibrosis. Moreover, the heterogeneity of the tumor microenvironment among different MPLC lesions poses a significant challenge to the efficacy of systemic therapies, including chemotherapy, targeted therapy, and immunotherapy [72,73].

In contrast, ablation therapy has attracted increasing attention as a promising modality due to its minimally invasive nature and favorable clinical outcomes. Since Dupuy’s initial report on lung cancer ablation in 2000 [74], this approach has evolved into a pivotal treatment option for early-stage lung cancer, particularly in patients unfit for surgical resection [75,76]. Technological advancements have significantly enhanced the safety, reproducibility, and efficacy of ablation, with 5-year overall survival rates approaching 95.00% and tumor-specific survival rates nearing 100.00% [77,78,79]. RFA, MWA, and CA remain the common employed modalities, all leveraging thermal energy to induce irreversible tumor cell destruction. CT-guided percutaneous ablation is currently the primary approach, though bronchoscopic techniques are gaining traction for their potential to minimize complications by avoiding pleural disruption. While RFA remains the most widely used modality, MWA offers advantages including a reduced heat-sink effect and more uniform energy distribution in lung tissue, whereas CA provides additional benefits including minimal procedural pain and real-time imaging for margin assessment [80,81,82]. The choice of ablation modality should be individualized based on lesion size and location, proximity to critical structures, comorbidities, and overall technical feasibility.

Ablation therapy has been increasingly utilized in the management of MPLC, demonstrating promising clinical outcomes [83,84]. Its minimally invasive nature, along with shorter recovery times and superior preservation of lung function, has positioned ablation as a viable treatment option for early-stage MPLC patients who are ineligible for surgery. Additionally, it serves as a valuable adjunct to surgical resection and expands therapeutic possibilities for patients with complex disease presentations [83,84,85]. Recent developments have introduced an integrated “one-stop” treatment model for MPLC, allowing surgical resection and ablation to be performed during a single hospitalization, and, in selected cases, under a single anesthesia session. This multidisciplinary strategy preserves lung parenchyma while enhancing treatment efficiency by reducing overall duration, healthcare costs, and perioperative psychological burden. As such, it offers a practical and patient-centered approach to the comprehensive management of MPLC.

A comprehensive evaluation of the included studies confirms the safety and feasibility of the combined surgery-ablation approach for MPLC. All included studies reported a 100.00% technical success rate for ablation, with no failures attributable to procedural or equipment-related issues. Meta-analysis revealed a pooled adverse event rate of only 14.23% (95% CI: 8.07–20.38%). While Harrison et al. [50] documented an adverse event rate of 50%, this was likely influenced by the small sample size (n = 4). In contrast, other studies reported complication rates below 30%, with most adverse events being mild, such as pneumothorax and subcutaneous emphysema, which resolved spontaneously. Severe complications, including pulmonary infection and hemoptysis, were rare and effectively managed with standard medical care. Importantly, no treatment-related mortality or life-threatening events were reported in any of the studies. The absence of complications in our case also supports the safety and feasibility of the combined therapy. Collectively, these findings indicate that the combined approach is technically reliable and clinically safe, warranting further investigation in larger prospective studies.

A review of the available studies suggests that the combined use of surgery and ablation for MPLC produces consistent treatment outcomes and prognoses. Meta-analysis showed a pooled ablation efficacy rate of 97.11% (95% CI: 85.81–100.00%). Regarding survival outcomes, three studies provided specific follow-up data. Harrison et al. [50] reported a median follow-up duration of 11 months, with no observed disease progression during this period. Liu et al. [49] conducted follow-up for a mean duration of 16 ± 13 months, during which the survival rate remained at 100%. Additionally, Xie’s study documented a 2-year local control rate of 71.4% and a median progression-free survival of 33 months [51]. The patient we reported remained alive after a 6-month follow-up period, with no local recurrence or distant metastasis. These findings suggest that the surgical-ablation strategy is both technically effective and associated with encouraging middle to long-term outcomes.

However, despite these encouraging results, several important limitations warrant cautious interpretation of the efficacy. First, most patients in available studies are retrospective collected with small sample sizes, while prospective studies are often limited by short follow-up durations and insufficient long-term outcome data. This limits the strength of clinical evidence. Second, in patients with multiple GGO, it is not feasible to biopsy every lesion before surgery, inevitably leading to the ablation of benign nodules and unnecessary injury to healthy lung tissue. Moreover, ablation efficacy is typically evaluated based on imaging changes rather than histopathological confirmation, which remains a key limitation when compared to surgical resection [86]. Additionally, although most included studies involved multiple GGO-dominant lesions—clinically more suggestive of MPLC—the diagnostic process distinguishing MPLC from IPM was not uniformly described, and molecular confirmation was rarely performed, representing another limitation of this review. Third, direct head-to-head comparisons between the combined surgery-ablation approach and other modalities (such as surgery alone, SBRT alone, or surgery + SBRT) are lacking; heterogeneous patient populations and variable endpoints further restrict generalisability. Therefore, while the meta-analysis provides a favourable safety-efficacy signal, we cannot definitively conclude that the combined approach is superior to other modalities.

In the future, large-scale prospective studies with long-term follow-up are urgently needed to validate the feasibility, safety, and survival benefits of combined surgery and ablation for MPLC across different tumor types, stages, and anatomical locations. In particular, randomized controlled trials are essential for direct comparisons of local tumor control, overall survival, and complication rates between modalities. The integration of ablation with systemic therapies also warrants further investigation. Cryoablation’s potential immunogenic effects may enhance responses to immune checkpoint inhibitors, while the optimal sequencing and selection criteria for combining ablation with neoadjuvant or adjuvant treatments such as Tyrosine Kinase Inhibitors (TKIs) remain undefined. In parallel, refining ablation protocols is crucial. Standardization of key technical parameters, including modality choice, energy settings, application duration, and imaging guidance, will help minimize complications and improve treatment consistency. Technological innovation, such as next-generation probes, enhanced energy control, synchronous biopsy capabilities, and more predictable ablation geometries, will further enhance precision and broaden clinical applicability. Addressing these issues will be critical for enhancing the clinical utility of ablation therapy and optimizing outcomes for MPLC patients.

## 6. Conclusions

This systematic review, along with a representative case, highlights the safety and efficacy of combining surgical resection with ablation in the management of MPLC. These findings support the adoption of individualized, multimodal strategies, particularly for patients with multiple or bilateral lesions who are unsuitable for extensive surgery, underscoring the value of integrating surgery and ablation as a unified therapeutic approach.

## Figures and Tables

**Table 1:** The major characteristics of studies on patients with multiple primary lung cancers treated with surgical and ablative therapies.

Author	Year	Country	Study Design	Study Period	Patients	Treatments	Procedure
Bao et al. [47]	2021	China	Prospective	June 2019–December 2020	5 solitary lung cancer and 10 MPLC.	Solitary lung cancer patients underwent MWA, and MPLC ones underwent VATS after MWA guided by ENB.	SuperDimension^TM^ navigation system; MWA: 40–80 W, 5–10 min; Surgery: VATS.
Zeng et al. [48]	2022	China	Retrospective	December 2019–June 2021	65 MPN patients.	8 patients only underwent MWA and 57 patients underwent VATS combined with MWA guided by ENB.	SuperDimension^TM^ navigation system; Median ablation power: 45 (30–70) W; Median ablation time: 3 (3–5) min.
Liu et al. [49]	2020	China	Retrospective	March 2015–March 2019	48 multifocal adenocarcinoma patients presenting GGO.	43 lesion wedge resections, 7 segmental resections and 17 lobectomy resections; 20 lesion ablations.	Thermal ablation: radiofrequency or microwave ablation. Ablation time: 30–120 min (mean: 43 min).
Harrison et al. [50]	2021	England	Retrospective	August 2018–January 2020	4 patients with multiple lung lesions	iCART	Neuwave percutaneous microwave ablation system; MWA: 60 W, 5 min; Surgery: uniportal VATS.
Xie et al. [51]	2022	China	Prospective	April 2018–July 2019	8 MPLC and 5 solitary lung cancer patients.	19 lesions were treated with ENB-guided MWA.	ENB or BTPNA; MWV: 50–80 W, 3–10 min.
Zhou et al. [52]	2023	China	Prospective	April 2015–December 2020	582 synchronous MPLC patients.	1198 lesion resections; CT-guided MWA treated 40 lesions in 37 patients; ENB-guided MWA treated 3 lesions in 3 patients.	Surgery: excision of primary and concurrent foci; “X”: including therapies like ablation and SBRT, management of high-risk residual, progressive, and new lesions.
Qu et al. [53]	2021	China	Retrospective	October 2015–December 2019	11 patients with multiple GGOs.	Surgical resection: primary lesion, invasive adenocarcinoma; Ablation: secondary, preinvasive or uncertain nature lesions.	ENB-guided MWA combined with single-port VATS; MWV: 60–80 W, 4–8 min.
Zarogoulidis et al. [54]	2023	Greece	Prospective	January 2019–July 2022	155 patients with single or multiple pulmonary nodules.	Using RFA to treat single or multiple nodules; All patients underwent pre-procedural PET-CT.	Radial-endobronchial ultrasound Fuji plus C-Arm (75 cases); Archemedes-Bronchus electromagnetic navigation system (80 cases).
Shan et al. [55]	2022	China	Retrospective	April 2022–July 2022	431 MPN patients.	The patients were divided into 4 groups: Group A (107 cases) underwent only CT-guided percutaneous biopsy; Group B (117 cases) was treated only with CT-guided thermal ablation; Groups C (103 cases) and D (104 cases) underwent CT-guided thermal ablation with immediate intraoperative biopsy.	CT-mediated percutaneous thermal ablation; MWV: 30–45 W, 2–5 min

Abbreviation: MPLCs: multiple primary lung cancers; MPNs: multiple primary nodules; MWA: microwave ablation; VATS: video-assisted thoracoscopic surgery; ENB: Electromagnetic navigation bronchoscopy; iCART: image-guided combined ablation and resection in thoracic surgery; BTPNA: bronchoscopic transparenchymal nodule access; GGOs: ground glass opacities; RFA: radiofrequency ablation.

**Table 2:** Outcomes and ablation effect indicators included in the study.

Author	Complications	Safety of Ablation	Ablation Efficacy	Incidence of Adverse Events	Follow-Up Period	Progression	Postoperative LOS
Bao et al. [47]	1 hemoptysis in patients with single lesion; 3 cases (air leakage, hemoptysis, lung infection) in patients with MPLC	100.00%	73.30%	26.67%	NA	NA	NA
Zeng et al. [48]	5 cases of pain, 2 of pneumothorax, 2 of subcutaneous emphysema, and 6 of persistent coughing.	100.00%	NA	23.08%	NA	No local recurrences or enlargement of pulmonary nodules.	Median: 8 days
Liu et al. [49]	2 cases of air leakage, 1 of chylothorax, and 1 of massive pleural effusion occurred after resection; 1 case of air leak after ablation.	100.00%	NA	7.14%	Mean: 16 ± 13 months (range: 5–60 months)	LTP rate: 0.00%	Surgery: 3–25 days (mean: 6.2 days); Ablation: 1–10 days (mean: 3.5 days)
Harrison et al. [50]	2 cases of intraoperative pneumothorax.	100.00%	100.00%	50.00%	Median: 11 months (range: 0–24 months)	No recurrence	NA
Xie et al. [51]	1 case of hemopneumothorax and 1 of pneumothorax.	100.00%	78.60%	10.50%	Median: 33 (95% CI: 30.6–35.4) months	2 year-LCR: 71.4%; Median PFS: 33 (95% CI: 15.0–51.0) months	NA
Zhou et al. [52]	2 cases of asymptomatic mild pneumothorax.	100.00%	100.00%	5.00%	3 months after ablation	LTP rate: 0.00%	NA
Qu et al. [53]	1 case of postoperative pneumothorax.	100.00%	100.00%	9.09%	Short-term	No local metastasis or recurrence	Mean: 6.2 ± 2.3 days
Zarogoulidis et al. [54]	Minor hemorrhage	100.00%	100.00%	NA	1 year	No recurrences	Radial-ebus ablation 1.6 days; Bronchus ablation: 1.4 days.
Shan et al. [55]	Group A: 7 cases of hemoptysis, 8 of pneumothorax and 4 of pleural effusion; Group B: 14 of pneumothorax and 7 of pleural effusion; Group C: 11 of pneumothorax and 5 of pleural effusion; Group D: 13 of pneumothorax and 9 of pleural effusion	100.00%	NA	18.20%	NA	NA	A: 5.20 ± 0.88 days; B: 4.96 ± 1.06 days; C: 5.09 ± 1.20 day; D: 5.07 ± 1.01 days.

Abbreviation: MPLCs: multiple primary lung cancers; NA: not applicable; LOS: length of stay; LTP: local tumor progression; PFS: progression-free survival; GGOs: ground glass opacities; LCR: local control rate.

**Table 3:** Common adverse events and complications in pulmonary nodule ablation.

Type	Main Cause	Management	Ablation Types
Pain	Thermal/cold stimulation near pleura or tissue tension	Intra-operation: opioids, sedatives; Post-operation: NSAIDs	RFA, MWA, CA
Post-ablation syndrome	Inflammatory cytokine release and necrotic absorption	Symptomatic care; Short-term low-dose corticosteroids	RFA, MWA
Cough	Thermal irritation to alveoli, bronchial/pleural membrane	Pre-operation: codeine; Post-operation: antitussives, antibiotics	RFA, MWA
Pleural reaction	Vagal nerve stimulation	Pause ablation; Atropine and sedatives	RFA, MWA
Pneumothorax	Lung puncture; emphysema/multiple passes	Often self-limited; Chest drainage, Pleurodesis	RFA, MWA, CA
Pleural effusion	Thermal injury reaction, lesion close to the pleura	Observation for mild cases; Drainage when symptomatic or large	RFA, MWA
Hemorrhage	Vascular injury during puncture or ablation	Hemostatics, lateral positioning, embolization or surgery	RFA, MWA, CA
Infection	Underlying lung disease; Immunosuppression; Multiple ablations	Prophylactic antibiotics; Abscess drainage	RFA, MWA, CA
Cavity formation	Expected course following necrotic tissue expulsion	Generally self-absorbed; Antibiotics and drainage if infected; Monitor for fungal infection	RFA, MWA
Skin frostbite	Probe proximity to skin; Frost formation on needle	Wound care, infection prevention	CA
Cold shock	Hypothermia near large vessels	Rewarming; Fluid resuscitation; Vasopressors	CA

Abbreviation: RFA: radiofrequency ablation; MWA: microwave ablation; CA: cryoablation.

## Data Availability

Data are contained within the article.

## Supplementary Materials

The following supporting information can be downloaded at: https://www.mdpi.com/article/10.3390/cancers17223699/s1. Search strategies; Table S1: Quality assessment of included studies. Figure S1: Risk of bias of included studies. Figure S2: Funnel plots of publication of bias of included studies. Primary outcomes were ablation efficacy (A) and incidence of adverse events (B).
