# Supplementary material for: Integrating Surgery and Ablative Therapies for the Management of Multiple Primary Lung Cancer: A Systematic Review
Source: Cancers (Basel). 2025 Nov 19;17(22):3699. doi: 10.3390/cancers17223699 (PMC12651476; doi:10.3390/cancers17223699)
Supplement: Supplementary file 1 [file cancers-17-03699-s001.zip › cancers-3778597-supplementary.pdf]

Integrating Surgery and Ablative Therapies for the Management of Multiple Primary Lung Cancer: A Systematic Review

Search strategies：

PubMed:

#1 "Multiple Pulmonary Nodule*"[Mesh] OR "Lung Neoplasms"[Mesh] OR "Multiple primary lung cancer*"[Title/Abstract] OR "Multiple primary lung neoplasm*"[Title/Abstract] OR MPLC[Title/Abstract] OR "Multiple lung tumor*"[Title/Abstract] OR "Multifocal lung cancer*"[Title/Abstract] OR "Multifocal lung tumor*"[Title/Abstract] OR "Multifocal pulmonary tumor*"[Title/Abstract] OR "Multifocal pulmonary cancer*"[Title/Abstract] OR "Synchronous lung cancer*"[Title/Abstract] OR "Synchronous lung tumor*"[Title/Abstract] OR "Metachronous lung cancer*"[Title/Abstract] OR "Metachronous lung tumor*"[Title/Abstract] OR "Second primary lung cancer*"[Title/Abstract] OR "Second primary lung tumor*"[Title/Abstract] OR "multiple isolated pulmonary nodule*"[Title/Abstract] OR "multiple solitary pulmonary nodule*"[Title/Abstract] OR "multifocal isolated lung nodule*"[Title/Abstract] OR "multiple discrete pulmonary nodule*"[Title/Abstract] OR SPN[Title/Abstract] OR "solitary pulmonary nodule*"[Title/Abstract]

#2 "Ablation technique*"[Mesh] OR "Radiofrequency Ablation"[Mesh] OR cryosurgery[Mesh] OR"Ablation therap*"[Title/Abstract] OR "interventional ablation*"[Title/Abstract] OR "percutaneous ablation*"[Title/Abstract] OR "image-guided ablation*"[Title/Abstract] OR "ablative therap*"[Title/Abstract] OR "thermal ablation*"[Title/Abstract] OR "Radiofrequency Ablation*"[Title/Abstract] OR "Radio-Frequency Ablation*"[Title/Abstract] OR RFA[Title/Abstract] OR "Radiofrequency coagulation*"[Title/Abstract] OR "Radio-frequency coagulation*"[Title/Abstract] OR "Radiofrequency electrocoagulation*"[Title/Abstract] OR "Radiofrequency thermocoagulation*"[Title/Abstract] OR "RF ablation*"[Title/Abstract] OR "Microwave* Ablation*"[Title/Abstract] OR "Microwave* coagulation*"[Title/Abstract] OR MWA[Title/Abstract] OR cryoablation*[Title/Abstract] OR "cryoablation therap*"[Title/Abstract] OR "hypothermal ablation*"[Title/Abstract] OR cryotherap*[Title/Abstract] OR "argon-helium cryoablation*"[Title/Abstract] OR "stereotactic ablative radiotherap*"[Title/Abstract] OR SABR[Title/Abstract]

#3 surger*[Title/Abstract] OR resection*[Title/Abstract] OR lobectomy[Title/Abstract] OR segmentectomy[Title/Abstract] OR "wedge resection"[Title/Abstract] OR pneumonectomy[Title/Abstract] OR "combined therap*"[Title/Abstract] OR thoracotomy[Title/Abstract] OR "video-assisted thoracic surgery"[Title/Abstract] OR VATS[Title/Abstract] OR "robot-assisted thoracic surgery"[Title/Abstract] OR RATS[Title/Abstract] OR "anatomical resection"[Title/Abstract] OR "minimally invasive surgery"[Title/Abstract]
#1 AND #2 AND #3

Embase:

#1 'multiple pulmonary nodule'/exp OR 'lung nodule'/exp OR 'lung cancer'/exp OR 'lung tumor'/exp OR 'multiple primary tumor'/exp OR 'multiple primary lung cancer*':ti,ab,kw OR 'multiple primary lung neoplasm*':ti,ab,kw OR MPLC:ti,ab,kw OR 'multiple lung tumor*':ti,ab,kw OR 'multifocal lung cancer*':ti,ab,kw OR 'multifocal lung tumor*':ti,ab,kw OR 'multifocal pulmonary tumor*':ti,ab,kw OR 'multifocal pulmonary cancer*':ti,ab,kw OR 'synchronous lung cancer*':ti,ab,kw OR 'synchronous lung tumor*':ti,ab,kw OR 'metachronous

lung cancer*':ti,ab,kw OR 'metachronous lung tumor*':ti,ab,kw OR 'second primary lung cancer*':ti,ab,kw OR 'second primary lung tumor*':ti,ab,kw OR 'multiple isolated pulmonary nodule*':ti,ab,kw OR 'multiple solitary pulmonary nodule*':ti,ab,kw OR 'multifocal isolated lung nodule*':ti,ab,kw OR 'multiple discrete pulmonary nodule*':ti,ab,kw

#2 ablation'/exp OR 'radiofrequency ablation'/exp OR 'microwave ablation'/exp OR 'cryosurgery'/exp OR 'cryotherapy'/exp OR 'ablation technique*':ti,ab,kw OR 'ablation therap*':ti,ab,kw OR 'interventional ablation*':ti,ab,kw OR 'percutaneous ablation*':ti,ab,kw OR 'image-guided ablation*':ti,ab,kw OR 'ablative therap*':ti,ab,kw OR 'thermal ablation*':ti,ab,kw OR ablation*:ti,ab,kw OR 'radiofrequency ablation*':ti,ab,kw OR 'radio-frequency ablation*':ti,ab,kw OR RFA:ti,ab,kw OR 'radiofrequency coagulation*':ti,ab,kw OR 'radio-frequency coagulation*':ti,ab,kw OR 'radiofrequency electrocoagulation*':ti,ab,kw OR 'radiofrequency thermocoagulation*':ti,ab,kw OR 'RF ablation*':ti,ab,kw OR 'microwave* ablation*':ti,ab,kw OR 'micro-wave* ablation*':ti,ab,kw OR 'microwave* coagulation*':ti,ab,kw OR 'micro-wave* coagulation*':ti,ab,kw OR MVA:ti,ab,kw OR cryosurger*:ti,ab,kw OR cryoablation*:ti,ab,kw OR 'cryoablation therap*':ti,ab,kw OR 'hypothermal ablation*':ti,ab,kw OR cryotherap*:ti,ab,kw OR 'argon-helium cryoablation*':ti,ab,kw OR 'stereotactic ablative radiotherap*':ti,ab,kw OR SABR:ti,ab,kw

#3 'surgery'/exp OR 'lung resection'/exp OR 'lobectomy'/exp OR 'segmentectomy'/exp OR 'pneumonectomy'/exp OR 'combined modality therapy'/exp OR surger*:ti,ab,kw OR resection*:ti,ab,kw OR 'surgical therap*':ti,ab,kw OR 'surgical resection*':ti,ab,kw OR lobectomy:ti,ab,kw OR segmentectomy:ti,ab,kw OR 'wedge resection':ti,ab,kw OR pneumonectomy:ti,ab,kw OR 'combined therap*':ti,ab,kw OR 'thoracic surgery':ti,ab,kw OR 'anatomical resection':ti,ab,kw OR VATS:ti,ab,kw

#1 AND #2 AND #3

Web of science:

#1 TS=("multiple pulmonary nodule*" OR "lung neoplasm*" OR "multiple primary lung cancer*" OR "multiple primary lung neoplasm*" OR MPLC OR "multiple lung tumor*" OR "multifocal lung cancer*" OR "multifocal lung tumor*" OR "multifocal pulmonary tumor*" OR "multifocal pulmonary cancer*" OR "synchronous lung cancer*" OR "synchronous lung tumor*" OR "metachronous lung cancer*" OR "metachronous lung tumor*" OR "second primary lung cancer*" OR "second primary lung tumor*" OR "multiple isolated pulmonary nodule*" OR "multiple solitary pulmonary nodule*" OR "multifocal isolated lung nodule*" OR "multiple discrete pulmonary nodule*" OR SPN OR "solitary pulmonary nodule*")

#2 TS=("ablation technique*" OR "radiofrequency ablation*" OR "cryosurgery*" OR "ablation therap*" OR "interventional ablation*" OR "percutaneous ablation*" OR "image-guided ablation*" OR "ablative therap*" OR "thermal ablation*" OR "radio-frequency ablation*" OR RFA OR "radiofrequency coagulation*" OR "radio-frequency coagulation*" OR "radiofrequency electrocoagulation*" OR "radiofrequency thermocoagulation*" OR "RF ablation*" OR "microwave* ablation*" OR "microwave* coagulation*" OR MWA OR "cryoablation*" OR "cryoablation therap*" OR "hypothermal ablation*" OR cryotherap* OR "argon-helium cryoablation*" OR "stereotactic ablative radiotherap*" OR SABR)

#3 TS=(surger* OR resection* OR lobectomy OR segmentectomy OR "wedge resection" OR pneumonectomy OR "combined therap*" OR thoracotomy OR "video-assisted thoracic surgery" OR VATS OR "robot-assisted thoracic surgery" OR RATS OR "anatomical resection" OR "minimally invasive surgery")

#1 AND #2 AND #3

Table S1 Quality assessment of included studies.

Author	Design	Q1	Q2	Q3	Q4	Q5	Q6	Q7	Q8	Score†
Bao et al.[48]	prospective	2	2	2	2	0	2	2	0	12
Zeng et al.[49]	retrospective	2	2	0	2	0	2	2	0	10
Liu et al.[50]	retrospective	2	2	0	2	0	2	2	0	10
Harrison et al.[51]	retrospective	2	2	0	2	0	2	2	0	10
Xie et al.[52]	prospective	2	2	2	2	0	2	2	2	14
Zhou et al.[53]	retrospective	2	2	0	2	0	2	2	0	10
Qu et al.[54]	retrospective	2	2	0	2	0	2	2	0	10
Zarogoulidis et al.[55]	prospective	2	2	2	2	0	2	2	0	12
Shan et al.[56]	retrospective	2	2	0	2	0	2	2	0	10

Q1: A clearly stated aim: the question addressed should be precise and relevant in the light of available literature.

Q2: Inclusion of consecutive patients: all patients potentially fit for inclusion (satisfying the criteria for inclusion) have been included in the study during the study period (no exclusion or details about the reasons for exclusion).

Q3: Prospective collection of data: data were collected according to a protocol established before the beginning of the study.

Q4: Endpoints appropriate to the aim of the study: unambiguous explanation of the criteria used to evaluate the main outcome which should be in accordance with the question addressed by the study. Also, the endpoints should be assessed on an intention-to-treat basis.

Q5: Unbiased assessment of the study endpoint: blind evaluation of objective endpoints and double-blind evaluation of subjective endpoints. Otherwise the reasons for not blinding should be stated.

Q6: Follow-up period appropriate to the aim of the study: the follow-up should be sufficiently long to allow the assessment of the main endpoint and possible adverse events.

Q7: Loss to follow up less than 5%: all patients should be included in the follow up. Otherwise, the proportion lost to follow up should not exceed the proportion experiencing the major endpoint.

Q8: Prospective calculation of the study size: information of the size of detectable difference of interest with a calculation of 95% confidence interval, according to the expected incidence of the outcome event, and information about the level for statistical significance and estimates of power when comparing the outcomes.

† The items are scored 0 (not reported), 1 (reported but inadequate) or 2 (reported and adequate).

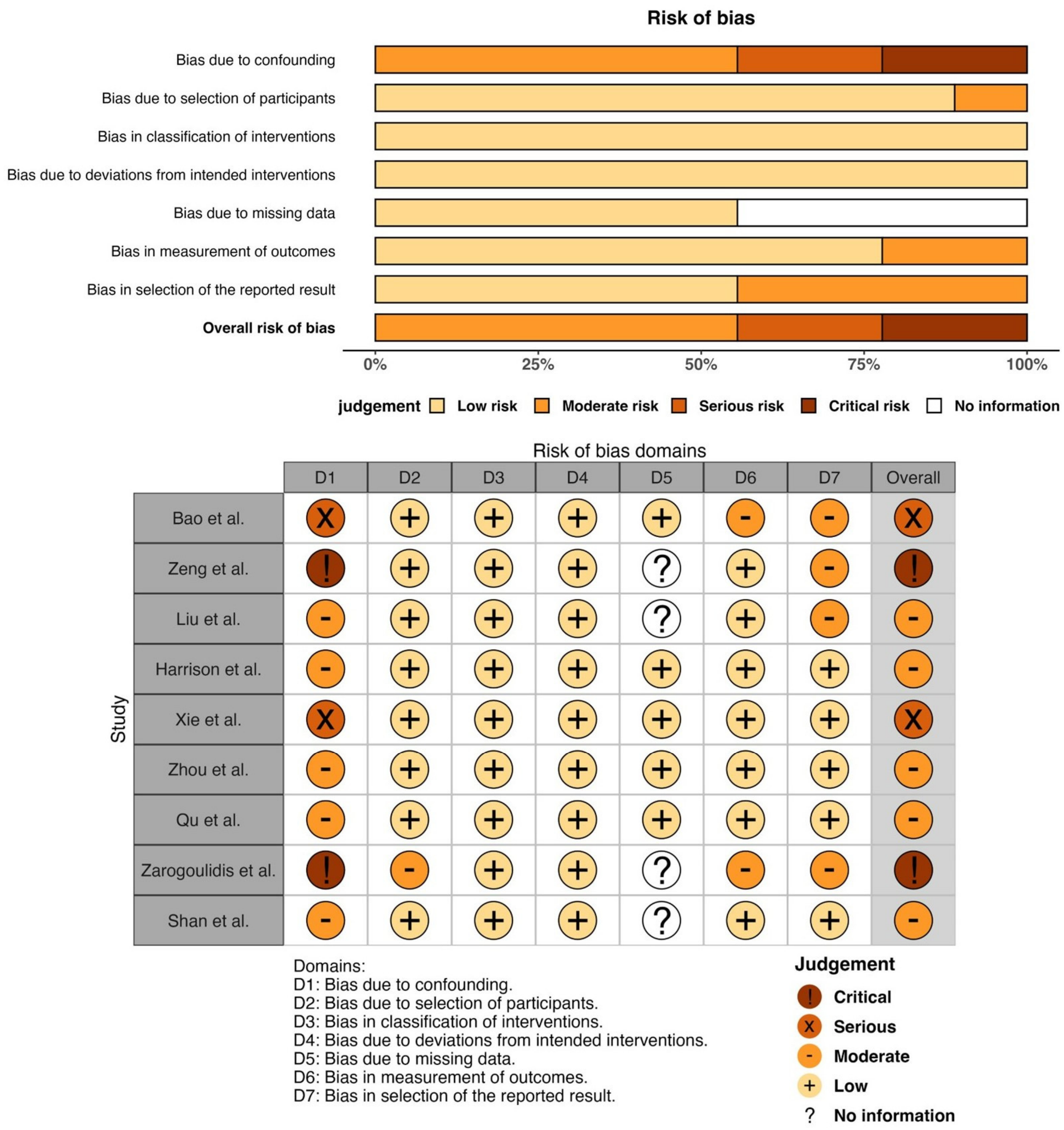

Figure S1 Risk of bias of included studies.

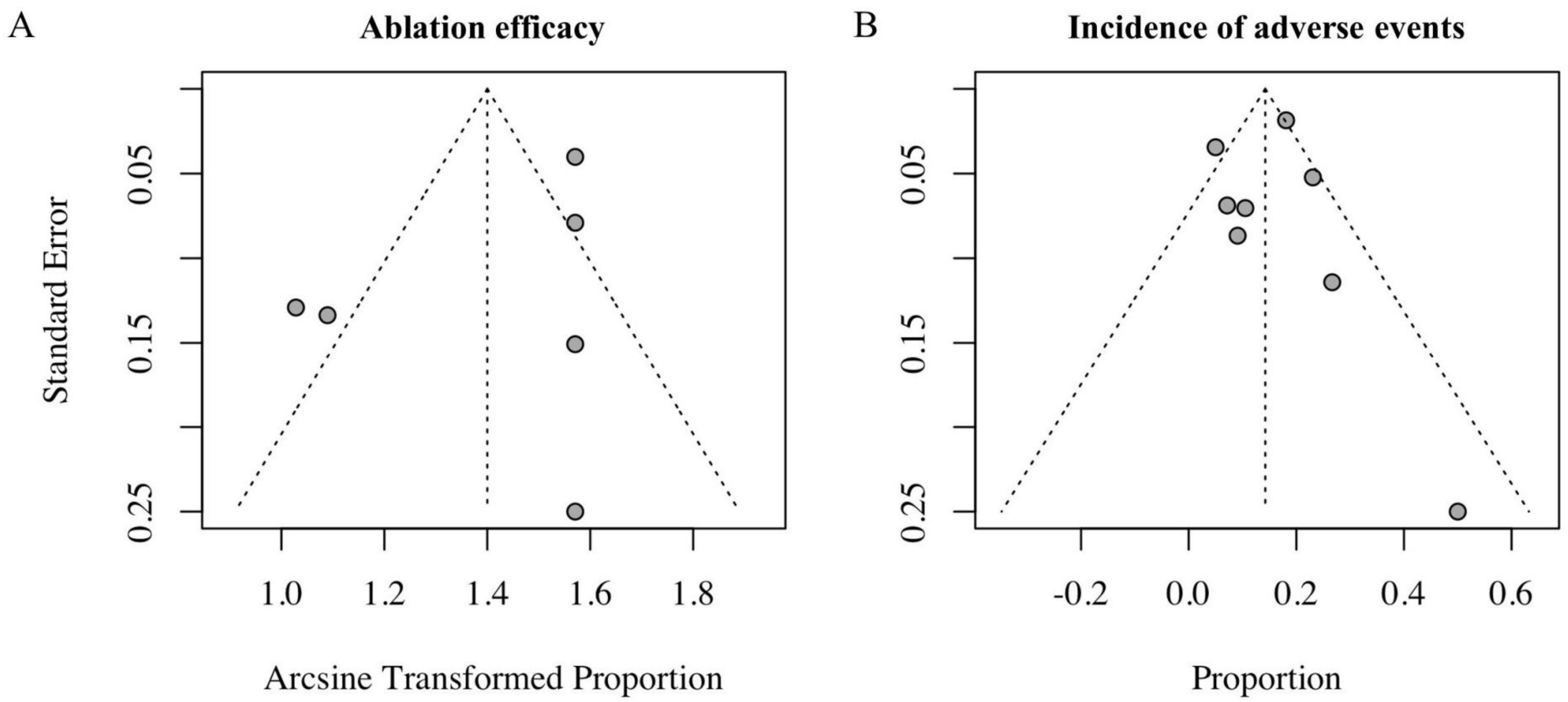

Figure S2 Funnel plots of publication of bias of included studies. Primary outcomes were ablation efficacy (A) and incidence of adverse events (B).
